# Understanding the Differences Between Online and Offline Mental Health Help Seekers: Cross-Sectional Comparative Study

**DOI:** 10.2196/69305

**Published:** 2025-11-07

**Authors:** Mohamed Adwi, Bahaa Mahmoud, Noha Amer, Roa Gamal Alamrawy, Ismail Sadek, Mohamed Elsheikh

**Affiliations:** 1 Shezlong Inc Giza Egypt; 2 Department of Psychiatry, Al-Azhar University Cairo Egypt; 3 Department of Psychiatry, Mansoura University Mansoura Egypt; 4 Ministry of Health and Population Mamoura Psychiatric Hospital, General Secretariat of Mental Health and Addiction Treatment Alexandria Egypt

**Keywords:** help-seeking behavior, online therapy, personality traits, self-stigma, telepsychiatry

## Abstract

**Background:**

Telepsychiatry has gained considerable attention, particularly during the COVID-19 pandemic. Although various factors influence the choice between online and offline modalities, differences among populations remain underexplored.

**Objective:**

This study aims to compare adults seeking mental health support online and offline in private clinics.

**Methods:**

In this cross-sectional study, we assessed differences in sociodemographic factors, internet accessibility and usability, previous help-seeking history, personality traits assessed using the Arabic Big Five Personality Inventory, and levels of self-stigma measured using the Self-Stigma of Seeking Help Scale.

**Results:**

In total, 259 participants were included (136 online and 123 offline). The online group had a higher proportion of university graduates (*P*=.02), employed individuals (*P*<.001), and those with better internet access (*P*=.03) and higher internet usability (*P*=.001). The offline group showed higher levels of conscientiousness (*P*=*.*003). The primary reasons for choosing online therapy were ease of access and time-saving. Logistic regression identified previous use of online psychiatry as the strongest factor associated with choosing online services (odds ratio [OR] 28.90, 95% CI 11.739-71.165; *P*<.001). Employment (OR 5.01, 95% CI 1.781-14.080; *P*=.002), better internet usability (OR 1.69, 95% CI 1.069-2.664; *P*=.03), and agreeableness (OR 1.16, 95% CI 1.001-1.351; *P*=.05) were also significant factors. In contrast, previous in-person visits (OR 0.11, 95% CI 0.048-0.269; *P*<.001), openness (OR 0.85, 95% CI 0.748-0.975; *P*=.02), and conscientiousness (OR 0.86, 95% CI 0.758-0.971; *P*=.02) were negatively associated with online preference.

**Conclusions:**

This study highlights key differences between online and offline mental health help seekers, enhances our understanding of treatment modality preferences, and paves the way for future research.

## Introduction

Telepsychiatry refers to the delivery of psychiatric assessment, consultation, and treatment services through telecommunication technologies that support real-time interactive communication between clinicians and patients. It is mostly implemented through videoconferencing, enabling direct face-to-face interactions at a distance. Online therapy, more broadly, encompasses any form of professional mental health support conducted over the internet, including text-, audio-, or video-based communication [1,2]. It has gained significant attention and adoption during the COVID-19 pandemic, which disrupted traditional face-to-face mental health service delivery [3]. Telepsychiatry has demonstrated the ability to perform various functions, such as monitoring, surveillance, mental health promotion, and treatment programs, as efficiently and effectively as in-person care [4].

Research on help-seeking preferences has revealed that several factors can influence individuals’ choices between online-based therapy and offline mental health help-seeking. Mental health literacy, gender, age, and location of residence have been identified as influential factors [5,6]. Online and offline users may differ in educational level, social support, social withdrawal, family attachment, and technological awareness [7]. Some individuals may lean toward face-to-face interactions, driven by personality traits and concerns about efficacy and privacy [8-10]. In contrast, online interventions are favored by many because they offer increased accessibility, overcoming barriers such as stigma, distance, and transportation issues [11-13].

Cross-cultural factors can significantly influence the preference for offline consultation or telepsychiatry; cultural background and identity can affect comfort with technology and acceptance of telepsychiatry [14]. Some patients prefer telepsychiatry, especially ethnic minorities and immigrants, to connect with mental health professionals who have the appropriate language and cultural background, regardless of location [15].

According to Andersen’s Behavioral Model of Health Services Use [16], the use of health services is shaped by 3 categories of factors, which can be applied to understand decisions about online mental health services. Predisposing factors, such as age, sex, and education, may influence individuals’ attitudes toward telepsychiatry. Enabling factors, including employment status, income, internet accessibility, and the ease of accessing therapists online, can facilitate or constrain the use of digital platforms. Need factors, such as privacy concerns and the preference for saving time, may further motivate individuals to choose online therapy over traditional in-person care. However, most studies comparing online and offline mental health help-seekers to better understand these factors have been conducted in Western populations, with few studies comparing populations from developed and developing countries [16-18]. Studies conducted in the Arab world, including Egypt, have primarily focused on readiness to use digital mental health services, as well as knowledge, attitudes, perceptions, and satisfaction with these services [19-21]. Nevertheless, there remains a gap in the literature addressing the factors that influence individuals’ choices between telepsychiatry and traditional in-person therapy in the Arab world [22].

Therefore, this study aims to address this gap by examining differences between online and offline mental health help seekers in Egypt, focusing on socioeconomic factors such as educational attainment, income level, and employment status, along with personality traits. Additionally, this study assessed differences with regard to the self-stigma of seeking help. In this study, we define “online help seekers” as individuals receiving therapy through videoconferencing platform, whereas “offline help seekers” are those attending in-person sessions at outpatient clinics. By undertaking this investigation, we aim to provide valuable insights into the field of telepsychiatry and mental health services.

## Methods

### Study Design and Participants

This cross-sectional study was conducted using a self-administered survey for both online and offline participants. Inclusion criteria were adult Egyptians aged 18 years or older, residing in Egypt, who sought mental health help either through an online telepsychiatry platform (Shezlong) or offline at private outpatient clinics, without psychotic features. To control for migration as a potential confounding factor, the study was limited to participants from a single country, ensuring that each participant could choose between online and offline therapy. To maintain comparability, only private clinics were selected for the offline group, aligning with the private nature of the online service. The study was guided by the CHERRIES (Checklist for Reporting Results of Internet E-Surveys) guidelines [23].

### Sample Size

The required sample size was determined using MedCalc version 18.2.1 (MedCalc Software Ltd). We based our calculation on a previous study that reported a mean agreeableness score of 4.67 (SD 1.17) among individuals preferring online mental health services and 5.13 (SD 1.10) among those preferring offline services [24]. Assuming a significance level α=.05, a power of 80%, and equal group allocation (1:1 ratio), the minimum sample size required was 190 participants. To ensure sufficient power and account for potential data loss, the sample size was rounded up to 200 participants, with 100 in each group.

### Recruitment

For online help seekers, a total of 7 therapists expressed interest in inviting their clients to participate. Each therapist sent a link to the online survey at the end of the session. For offline participants, a total of 6 therapists working in private clinics provided a hard copy of the survey following clinic consultations. A consent form was placed on the first page of the online survey, and only participants who provided consent proceeded to the subsequent pages, where they answered the age question to confirm eligibility based on predetermined inclusion criteria. Due to cultural sensitivity toward written consent, initial oral consent was obtained from offline participants, after which those who agreed to participate completed a paper-based survey that began with a written consent statement. Data were collected randomly from January to June 2024. To minimize selection bias, therapists were instructed to approach all eligible clients during the recruitment period.

There are notable sociodemographic differences between public and private sector help-seekers in Egypt. Additionally, there is no equivalent public sector online mental health service with comparable reach or accessibility, as the only existing platform remains in a limited beta phase. Moreover, the private sector accounts for a substantial portion of outpatient mental health care use, even among lower-income individuals, due to perceptions of better quality and accessibility compared with public services. Therefore, we focused exclusively on the private sector for the comparison between online and offline help-seekers [25].

### Data Collection Tools

The participants completed a self-administered questionnaire. The overall instrument demonstrated acceptable internal consistency in our sample (Cronbach α=0.674). The questionnaire consisted of the following sections: demographic and social factors, internet accessibility and usability, previous history of online or offline help seeking, reasons for preferring online therapy, the Arabic Big Five Personality Inventory, and the Self-Stigma of Seeking Help (SSOSH) scale.

#### Demographic and Socioeconomic Factors

Demographics included age, sex, educational status, last educational attainment, and marital status. Other variables included residence, monthly salary, breadwinner status, and receiving charity. Participants who were employed were asked about employment type (part time or full time), work shift (rotational or fixed), working location, salary range, and work sector.

#### Internet Accessibility and Usability

Internet accessibility was assessed as yes or no, and internet usability was measured on a Likert scale from 1 to 5, where 1 represents the lowest and 5 the highest.

#### Previous History of Online or Offline Help Seeking

History of online or offline help seeking was assessed by asking about previous experience with offline help seeking through clinics or online through videoconference, with a yes or no response.

#### Reasons for Preferring Online Therapy

Online participants were asked to rate the strength of each reason for preferring online therapy using a Likert scale, where 0=no, 1=moderate, and 2=strong. The options were: ease and comfort, saving time, cost savings, feeling of confidentiality, lack of nearby therapists, availability of many therapists to choose from, preferred therapist is available online only, and others (please specify).

#### The Arabic Big Five Personality Inventory

A 25-item questionnaire was used to assess the 5 major personality traits: neuroticism (N), extraversion (E), agreeableness (A), openness to experience (O), and conscientiousness (C). Sample items include: “I am a sociable person,” “I am an anxious person,” “I like new ideas,” and “I am organized and disciplined.” The reliability of this inventory ranged between 0.70 and 0.83, while validity ranged from 0.49 to 0.86 [26,27]. Respondents rated each item on a 4-point Likert scale: 1=no, 2=some, 3=much, and 4=always. Each trait’s total score ranges from 5 to 20, with higher scores reflecting stronger expression of that trait. In the current sample, internal consistency was assessed using Cronbach α. The Arabic Big Five Personality Inventory demonstrated acceptable overall reliability (α=0.658). Subscale reliability coefficients were as follows: extraversion (α=.790), neuroticism (α=0.757), agreeableness (α=0.909), openness (α=0.839), and conscientiousness (α=0.820).

#### SSOSH Scale

The SSOSH consists of 10 items on a Likert scale from 1 to 5, where 1=strongly disagree and 5=strongly agree. Items 2, 4, 5, 7, and 9 are reverse scored, and higher scores indicate greater of self-stigma [28]. Sample items include: “I feel inferior if I go to a therapist for help” and “Seeking help from a professional does not threaten my self-confidence.” The translated Arabic version is available on the official website of the Self Stigma Research Collaborative at Iowa State University [29]. In the current sample, Cronbach α for the SSOSH was 0.398, indicating low internal consistency.

### Statistical Analysis

The collected data were wrangled, coded, and analyzed using SPSS software (version 25; IBM Corp) and R (R Foundation for Statistical Computing) to generate a correlation heatmap of the Big Five traits and SSOSH scale. Quantitative variables are expressed as mean (SD), whereas categorical variables are expressed as n (%). An independent 2-tailed *t* test was used to estimate differences in continuous variables between the online and offline groups, whereas the chi-square test and Monte Carlo exact probability were used to assess the difference in categorical variables, with column proportions analyzed for significant variables. Pearson correlation was used to assess the relationship between the Big Five traits and the SSOSH scale. A multivariable logistic regression model was conducted using important significant variables, the Big Five traits, and the SSOSH scale to identify the significant factors associated with preference for online consultations. Odds ratios (ORs) and 95% CIs were reported. Statistical significance was set at *P*<.05. Cronbach α values were calculated to evaluate the internal consistency of the psychometric scales used in the current sample.

### Ethical Considerations

Ethics approval was obtained from the Ethics Committee of Al-Azhar Faculty of Medicine (registry number Psych._84 Med.Research._Prevalence Depression/Pts.-MS._00000084). All procedures involving human participants were conducted in accordance with the ethical standards of the institutional research committee of Al-Azhar Faculty of Medicine and the 1964 Helsinki Declaration and its later amendments or comparable ethical standards. The process of obtaining informed consent, described above, was implemented for both online and offline participants using electronic and written formats, respectively. Participants’ privacy and confidentiality were safeguarded throughout all study phases, with anonymized data used for analysis and reporting. Online participants were offered a 10%-20% discount for a future online therapy session as a token of appreciation, whereas offline participants received no compensation.

## Results

### Demographic Characteristics

A total of 304 eligible participants were approached, of whom 259 agreed to participate, yielding a response rate of 85.2%. The study included 259 participants, with 136 in the online group and 123 in the offline group. The majority of participants were female, representing 80.9% (110/136) in the online group and 73.2 % (90/123) in the offline group. The mean age was similar between groups, with the online group having a mean age of 28.92 (6.35) years and the offline group having a mean age of 29.21 (9.55) years.

Significant differences were observed in the current educational level between the 2 groups (*P*=.003). The online group had a lower percentage of students (22/136, 16.2%) compared to the offline group (35/123, 28.5%) but a higher proportion of graduates (114/136, 83.8%) compared to 68.3% (84/123). When considering the highest level of education achieved, the offline group had a higher proportion of participants with secondary education or less (16/119, 13.4%) compared to the online group (6/137, 4.4%), whereas the online group had more participants with a university degree (109/136, 80.1%) compared to 68.1% (81/119), a statistically significant difference (*P*=.02). There were no significant differences in marital status between the groups, with the majority of participants being single, particularly in the online group (96/136, 70.6%) compared to 61.8% (76/123; Table 1).

**Table 1:** Demographic characteristics.

Variable		Online group (n=136)	Offline group (n=123)	Test statistic		Effect size		*P* value	
**Sex**					2.2 (1)^a^		0.092^b^		.14
	Male, n (%)	26 (19.1)	33 (26.8)						
	Female, n (%)	110 (80.9)	90 (73.2)						
Age in years, mean (SD)		28.92 (6.35)	29.21 (9.55)	–0.284 (251)^c^		0.0358^d^		.78	
**Current education level, n (%)**					—^e^		0.205^b^		.003^f^
	Not educated^g^	0 (0)	4 (3.3)						
	Student^g^	22 (16.2)	35 (28.5)						
	Graduate^g^	114 (83.8)	84 (68.3)						
**Highest education level achieved (n=256), n (%)**					7.6 (2)^a^		0.173^b^		.02
	Secondary or less^g^	6 (4.4)	16 (13.4)						
	University degree^g^	109 (80.1)	81 (68.1)						
	Higher education^h^	21 (15.4)	22 (18.5)						
**Marital status, n (%)**					3.5 (3)^a^		0.116^b^		.32
	Single	96 (70.6)	76 (61.8)						
	Married	35 (25.7)	38 (30.9)						
	Divorced	5 (3.7)	8 (6.5)						
	Widow	0 (0.0)	1 (0.8)						

^a^Chi-square test (degrees of freedom).

^b^Cramer V.

^c^2-tailed *t* test (degrees of freedom).

^d^Cohen *d*.

^e^Not applicable.

^f^Monte Carlo exact probability.

^g^Significant difference between both groups.

^h^No significant difference between both groups.

### Social and Economic Characteristics

The social and economic characteristics of the participants revealed notable differences between the online and offline groups. A significant difference was observed in working status, with a higher proportion of participants in the online group being employed (105/136, 77.2%) compared to the offline group (69/123, 56%), indicating a difference of 21.2% (*P*<.001). This association reflected a moderate effect size (Cramer V=0.224). Both groups predominantly resided in urban areas, with the online group showing 96.3% (131/136) compared to 91.1% (112/123) in the offline group, although this difference was not statistically significant. The proportion of participants who were breadwinners was also comparable between the groups, with 15.4% (21/136) in the online group and 22.8% (28/123) in the offline group.

Working conditions indicated that both groups had similar distributions, with the majority working full-time, and there were no significant differences in workplace (office-based vs remote) or working time (fixed vs rotational shifts). Participants were engaged in various sectors, including health, technology, communication, governmental services, and entrepreneurship. Income levels also showed no significant differences, with 22.3% (23/103) of the online group earning more than 20,000 Egyptian pounds (US $419.03) compared with 25.8% (17/66) in the offline group (*P*=.25) (Table 2).

**Table 2:** Social and economic characteristics. A currency exchange rate of EGP £1=US $0.02078 is applicable.

Variable			Online group (n=136), n (%)		Offline group (n=123), n (%)		Chi-square (*df*)			Effect size, Cramer V			*P* value	
**Residence**								3.1 (1)			0.109			.08
	Urban	131 (96.3)		112 (91.1)										
	Rural	5 (3.7)		11 (8.9)										
**Breadwinner**								2.3 (1)			0.093			.13
	Yes	21 (15.4)		28 (22.8)										
	No	115 (84.6)		95 (77.2)										
**Receiving pocket money**								2.1 (1)			0.090			.15
	Yes	52 (38.2)		58 (47.2)										
	No	84 (61.8)		65 (52.8)										
**Working status**								13.1 (1)			0.224			<.001
	Yes^a^	105 (77.2)		69 (56.1)										
	No^a^	31 (22.8)		54 (43.9)										
**Working condition (n=174)**								0.039 (1)			0.015			.84
	Part-time	26 (24.8)		18 (26.1)										
	Full-time	79 (75.2)		51 (73.9)										
**Working place (n=174)**								1.8 (2)			0.102			.41
	Remote or home	27 (25.7)		17 (24.6)										
	Office based	45 (42.9)		36 (52.2)										
	Both	33 (31.4)		16 (23.2)										
**Working time (n=172)**								0.019 (1)			0.011			.89
	Fixed shifts (9 AM to 5 PM)	60 (57.1)		39 (58.2)										
	Rotational shifts	45 (42.9)		28 (41.8)										
**Working sector (n=169)**								6.9 (5)			0.202			.23
	Health	18 (18.0)		14 (20.3)										
	Technology	29 (29.0)		15 (21.7)										
	Communication	7 (7.0)		10 (14.5)										
	Governmental or public services	8 (8.0)		3 (4.3)										
	Entrepreneurship	7 (7.0)		10 (14.5)										
	Other	31 (31.0)		17 (24.6)										
**Income in Egyptian pounds (n=169)**								5.4 (4)			0.179			.25
	Less than 5000	17 (16.5)		14 (21.2)										
	5000-10,000	34 (33.0)		21 (31.8)										
	10,000-15,000	20 (19.4)		5 (7.6)										
	15,000-20,000	9 (8.7)		9 (13.6)										
	More than 20,000	23 (22.3)		17 (25.8)										

^a^Significant difference between both groups.

### Internet Access

Regarding internet access, the online group demonstrated a higher connectivity rate, with 97.8% (133/136) having internet access compared to 91.9% (113/123) in the offline group (*P*=.03). Additionally, the online group reported better internet usability, with a mean score of 4.57 (0.64) compared to 4.14 (1.25) in the offline group, indicating a significant difference (*P*=.001) (Table 3).

**Table 3:** Internet access and history of psychiatric visit.

Variable			Online group (n=136)		Offline group (n=123)		Test statistic		Effect size, Cramer V		*P* value
**Having internet access, n (%)**							4.8 (1)^a^		0.135		.03
	Yes^b^	133 (97.8)		113 (91.9)							
	No^b^	3 (2.2)		10 (8.1)							
Internet usability, mean (SD)			4.57 (0.641)		4.14 (1.25)		3.515 (257)^c^		0.295		.001
**History of psychiatric visit, n (%)**							35.8 (1)^a^		0.372		<.001
	Yes^b^	49 (36.0)		90 (73.2)							
	No^b^	87 (64.0)		33 (26.8)							
**Online through video, n (%)**							100.7 (1)^a^		0.623		<.001
	Yes^b^	107 (78.7)		20 (16.3)							
	No^b^	29 (21.3)		103 (83.7)							

^a^Chi-square test (degrees of freedom).

^b^Significant difference between both groups.

^c^2-tailed *t* test (degrees of freedom).

### History of Psychiatric Visit

This study examined the history of psychiatric visits for each group. The offline group had a 37.2% higher rate of clinic visits, with almost three-quarters reporting such visits (90/123, 73%) compared to only 36% (49/136) in the online group (*P*<.001). Similarly, the online group demonstrated a significantly higher use of online video consultations, with 78.7% (107/136) receiving this service compared to only 16.3% (20/123) in the offline group (*P*<.001). This finding was associated with a large effect size (Cramer V=0.623) (Table 3).

### Reasons for Choosing Online Therapy

**Figure 1:**
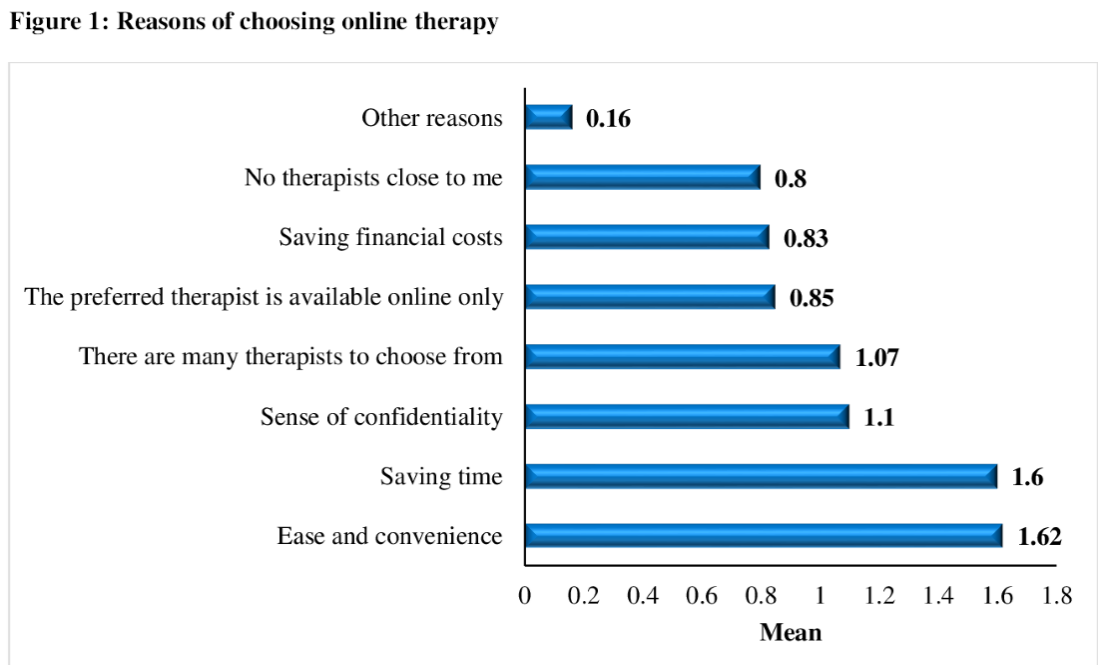
Reasons of choosing online therapy.

Figure S1 in Multimedia Appendix 1 shows the primary reasons participants chose online therapy, with the top 4 factors being ease and convenience (mean 1.62, SD 0.55), saving time (mean 1.6, SD 0.58), sense of confidentiality (mean 1.1, SD 0.82), and availability of many therapists (mean 1.07, SD 0.76; Figure 1).

### Big Five Personality Trait

With regard to personality traits, the only statistically significant difference between the groups was found in conscientiousness, with the offline group scoring higher than the online group (mean 13.44, SD 3.47 vs mean 12.15, SD 3.47; *P*=.003), reflecting a moderate effect size (Cohen *d*=0.37). No significant differences were observed for the other personality traits, including extraversion, neuroticism, agreeableness, and openness, which showed similar levels between the 2 groups. Among the personality traits, agreeableness had the highest mean in both groups. In the online group, openness was the second highest, whereas conscientiousness was the second highest in the offline group (Table 4).

**Table 4:** Big Five personality traits and the Self-Stigma of Seeking Help (SSOSH).

Variable	Online group (n=136), mean (SD)	Offline group (n=123), mean (SD)	2-tailed *t* test (*df*)	Effect size, Cohen *d*	*P* value
Extraversion	11.37 (2.79)	11.98 (3.27)	–1.635 (257)	0.201	.10
Neuroticism	12.57 (3.04)	12.74 (3.16)	–0.431 (257)	0.055	.67
Agreeableness	15.18 (3.51)	15.45 (3.12)	–0.635 (257)	0.081	.53
Openness	12.84 (3.71)	13.04 (3.68)	–0.440 (257)	0.054	.66
Conscientiousness	12.15 (3.47)	13.44 (3.5)	–2.982 (257)	0.37	.003
SSOSH	19.96 (5.68)	22.96 (6.48)	–3.973 (257)	0.492	<.001

### SSOSH

The offline group showed a higher level of SSOSH, with a mean score of 22.96 (6.48) compared to the online group (mean 19.96, SD 5.68), reflecting a significant difference (2-tailed t_257_=–3.973; *P*<.001) and a moderate-to-large effect size (Cohen *d*=0.492) (Table 4). We examined the correlation between personality traits and SSOSH in both the online and offline groups. Several significant correlations were identified in the online group. Extraversion showed a weak negative correlation with SSOSH (*r*=–0.199; *P*=.02), while openness exhibited a stronger negative correlation (*r*=–0.287; *P*=.001). Additionally, conscientiousness was also negatively correlated with SSOSH (*r*=*-*0.192; *P*=.03) (Table 4).

In the offline group, openness was the only trait that significantly correlated with SSOSH (*r*=–0.226; *P*=.12). These findings suggest that openness is consistently associated with lower self-stigma across both groups, whereas extraversion and conscientiousness appear to play more prominent roles in the online group (Table 5).

**Table 5:** Correlation between the Big Five traits and the Self-Stigma of Seeking Help (SSOSH) scale among online and offline groups (Pearson r and *P* value).

Group			Extraversion		Neuroticism		Agreeableness		Openness		Conscientiousness
**Online (n=136)**											
	*r*	–0.199		0.149		–0.143		–0.287		–0.192	
	*P* value	.02		.08		.10		.001		.03	
**Offline (n=123)**											
	*r*	–0.083		0.140		–0.052		–0.226		–0.113	
	*P* value	.36		.12		.57		.01		.21	

To account for the increased risk of type I error due to multiple comparisons, the Benjamini–Hochberg procedure was applied to control the false discovery rate at 0.05 across bivariate tests. Seven variables remained statistically significant after correction: SSOSH scores, history of psychiatric visits (both clinic-based and online), working status, ease of internet access, current education level, and conscientiousness. Other variables, including highest level of education and internet access, did not remain significant after correction (Table S1 in Multimedia Appendix 1).

### Logistic Regression Analysis of Factors Affecting Choosing Online Sessions

Logistic regression analysis revealed several significant factors associated with choosing online mental health services. Multicollinearity among predictors was assessed using variance inflation factors; all values were below 1.52, indicating an acceptable level of collinearity (Table S2 in Multimedia Appendix 1). The model demonstrated a good fit, as indicated by a nonsignificant Hosmer–Lemeshow test (*χ*²_8_=3.9; *P*=.86), and explained a high proportion of variance in the outcome (Nagelkerke *R*²=0.662). After adjustment for confounders, a history of online psychiatric consultation was the strongest factor (OR 28.90, 95% CI 11.739-71.165; *P*<.001), followed by a history of in-person psychiatric consultation, which was negatively associated with choosing online services (OR 0.11, 95% CI 0.048-0.269; *P*<.001). Being employed (OR 5.01, 95% CI 0.327-2.739; *P*=.002) and higher internet usability (OR 1.69, 95% 0.445-56.969; *P*=.03) were also significant factors. Among the personality traits, higher agreeableness (OR 1.16, 95% CI 1.001-1.351; *P*=.05) was positively associated with choosing online therapy, while higher openness (OR 0.85, 95% CI 0.748-0.975; *P*=.02) and conscientiousness (OR 0.86, 95% CI 0.758-0.971; *P=*.02) were negatively associated. Other factors such as education level, internet access, and SSOSH scores were not significant (Table 6). A marginal OR plot was constructed to show the adjusted effects of the statistically significant factors identified in the logistic regression model (Figure S2 in Multimedia Appendix 1).

**Table 6:** Logistic regression analysis of factors affecting choice of online sessions (overall percentage=85.7%; χ²13=177.5 and *P*<.001; Hosmer–Lemeshow test χ²8=3.9 and *P*=.86; Nagelkerke R²=66.2%).

Independent variable		Unstandardized β coefficient	Unstandardized SE	OR^a^	(95% CI)	*P* value
Constant		–0.231	2.165	0.794		.92
**Education level^b^**						>.99
	Illiterate	–17.562	14784.757	0.000	0.000	>.99
	Student	–0.056	0.543	0.946	0.327-2.739	.92
Working status^c^		1.611	0.527	5.007	1.781-14.080	.002
Having internet access^c^		1.617	1.238	5.037	0.445-56.969	.19
Internet usability^c^		0.523	0.233	1.688	1.069-2.664	.03
History of psychiatric consultation (clinic)^c^		–2.178	0.442	0.113	0.048-0.269	<.001
History of psychiatric consultation (online)^c^		3.364	0.460	28.904	11.739-71.165	<.001
Extraversion		–0.091	0.070	0.913	0.796-1.048	.20
Neuroticism		–0.076	0.070	0.927	0.808-1.063	.28
Agreeableness		0.151	0.076	1.163	1.001-1.351	.048
Openness		–0.158	0.068	0.854	0.748-0.975	.02
Conscientiousness		–0.153	0.063	0.858	0.758-0.971	.02
SSOSH^d^		–0.056	0.034	0.946	0.885-1.011	.10

^a^OR: odds ratio.

^b^Reference: graduate.

^c^Reference: no.

^d^SSOSH: Self-Stigma of Seeking Help.

## Discussion

### Overview

The internet has become increasingly important as primary channel for delivering remote therapy. Over the past 20 years, there has been significant growth in online platforms supporting individuals with various mental health conditions [30,31]. However, research has primarily focused on comparing the efficacy of telepsychiatry with offline methods [32,33], with less emphasis on the individual factors that influence the choice of online options. The personal and psychological factors associated with choosing online therapy over offline therapy remain insufficiently explored.

This study aims to provide preliminary data on the differences between individuals who prefer online versus offline mental health services, with a focus on the relationships between sociodemographic characteristics, previous mental health experiences, personality traits, self-stigma, and the preferred modality of help-seeking. Multivariable logistic regression analysis identified a history of online psychiatric consultation as the strongest factor associated with online preference, followed by employment, ease of internet use, and agreeableness. Conversely, previous offline psychiatric visits, along with higher levels of openness and conscientiousness, were significantly associated with a preference for offline care.

We found that the online group was more likely to be graduates, employed, hold a university degree, and have greater internet access and usability. This suggests that telepsychiatry may be more widely adopted when internet access is better and education levels are higher. A national survey in Egypt found that individuals with higher education and current employment status demonstrated greater awareness of and a more positive attitude toward telemedicine [34], aligning with broader research that links telemedicine use to higher educational levels, better internet access, and greater digital literacy [35,36]. Another study on Egyptian students reported that unfamiliarity with electronic mental health is among the most commonly perceived barriers [37]. Additionally, graduates and employed individuals often face time constraints and must balance multiple responsibilities, making the flexible nature of online therapy an appealing option.

This study revealed no significant difference in income levels between the online and offline groups. Globally, individuals with lower income levels face greater challenges in accessing mental health services [38]. However, participants in both groups in this study had already accessed private mental health care, suggesting that they likely have the financial resources to afford such services, which may minimize the impact of income disparities between the groups. In addition, several other factors influence the decision to seek mental health care in the Arab world, including mental health stigma, negative perceptions and attitudes toward mental illness, low mental health literacy, family dynamics, structural barriers related to the health care system, and service accessibility [39,40]. Such factors have also been identified in studies conducted in Egypt, where help-seeking behaviors are often affected by strong social stigma and reliance on informal support [41,42].

We found that more than 90% of both the offline and online groups reside in urban areas, indicating that rural residents are less likely to seek mental health help, whether online or offline. Egyptians living in rural areas tend to have lower levels of mental health literacy, higher levels of stigma, and limited internet access, all of which may contribute to reduced help-seeking rates compared with urban residents [43-45]. Additionally, there is an unequal distribution of mental health services across Egypt, where the majority of psychiatric facilities and specialists are concentrated in urban centers. As a result, individuals living in rural areas face limited access to mental health care, both online and offline [46]. This pattern is commonly seen in other countries as well; rural populations often face logistical challenges, including a shortage of mental health professionals, significant distances to health care facilities, and lower levels of mental health literacy, which hinder their ability to recognize mental health issues and the importance of seeking help [47-49].

This study found that the offline group had a statistically significant association with previous clinic visits, whereas the online group had a similar association with use of video-based online therapy. Notably, this was the strongest factor associated with choosing online therapy in the logistic regression, with OR 28.90 (95% CI 11.739-71.165). This pattern may be explained by the mere exposure effect, whereby individuals develop a preference or bias toward familiar options due to increased comfort, perceived safety, and reduced cognitive effort [50]. Previous literature suggests that when individuals have experience using online services, they are more likely to prefer these platforms when seeking help for mental health problems [10,51]. However, choosing face-to-face counseling over online therapy may be attributed to unfamiliarity with online counseling and concerns about privacy and security [52,53]. Additionally, the association may reflect the persistence of the initial factors that led individuals to choose online therapy, such as the need for privacy, scheduling flexibility, time efficiency, or work-related constraints.

The strongest reasons for choosing online therapy over offline were ease and convenience, time saving, and a greater sense of confidentiality. This trend is common, as online therapy is perceived as beneficial due to its flexibility, allowing clients to schedule sessions without needing to travel, saving both time and money [54,55]. This convenience is especially helpful for individuals balancing work, family, or other commitments [56]. Additionally, confidentiality is a primary motive for choosing online therapy, as it offers greater privacy, especially for individuals dealing with stigmatized issues [57].

Agreeableness was the most prevalent trait in both groups in this study. Agreeable individuals are more likely to have a positive attitude toward seeking professional help and recognize the need for information and treatment [55,56], with a lower likelihood of holding stigmatizing beliefs about mental illness [57]. In many Arab countries, patients tend to favor a paternalistic model of the doctor-patient relationship, where the physician is expected to take the lead in decision-making, with little involvement from the patient, a tendency that aligns with a high level of agreeableness [58,59]. Moreover, high agreeableness has been commonly observed in studies conducted on Arab populations, including Egyptians, reflecting cultural values of collectivism and social conformity [58-60].

The logistic regression analysis in this study identified associations between specific personality traits and the preference for telepsychiatry. Higher levels of agreeableness showed a marginal association with choosing online therapy, while higher levels of openness and conscientiousness were negatively associated. Previous studies have shown that individuals high in agreeableness may demonstrate greater acceptance of online therapy and a higher capacity to adapt to new situations that require online therapy [61,62]. Moreover, their heightened sensitivity to social evaluation from others may make them prefer the privacy offered by telepsychiatry, as accessing care from home helps minimize exposure to potential stigma associated with attending in-person clinics [63].

Unexpectedly, higher openness was negatively associated with the preference for telepsychiatry. While openness is generally linked to creativity and a willingness to explore new experiences [64,65], individuals high in this trait may favor offline therapy, potentially due to its greater opportunity for in-depth emotional connection and self-disclosure [66]. This aligns with clinical observations from Shezlong, where many clients tend to keep their cameras off during initial video sessions—an act that may reflect lower levels of openness.

Conscientiousness was statistically higher in the offline group and negatively associated with telepsychiatry. This trait is associated to a strong sense of responsibility, self-discipline, and a goal-oriented mindset [67]. Seeking help in the clinic may require higher levels of commitment, punctuality, and adherence to structured appointments compared with the flexibility of online therapy. Currently, the literature lacks direct studies on personality traits and telepsychiatry use. Most research focuses on broader help-seeking behaviors or general telehealth adoption [68,69].

Self-stigma was higher among the offline group. Previous research has demonstrated that higher levels of self-stigma are associated with a reduced likelihood of seeking offline psychological help [70,71]. A systematic review conducted in the Middle East found that both public and self-stigma serve as major barriers to accessing mental health care, with many individuals turning to family members or traditional healers before seeking professional help [67]. Addressing these cultural nuances through targeted awareness campaigns and community-based initiatives may help reduce stigma and promote help-seeking behavior across age groups [72,73].

Although self-stigma was lower among the online group, it was not independently associated with the choice of online help when controlling confounding variables in the logistic regression analysis. The relation between self-stigma and help seeking has not been sufficiently investigated in the context of online help-seeking or telepsychiatry. A previous study from China reported a negative association between self-stigma and the willingness to seek online help, though it did not include a comparison group of offline help-seekers [70]. Another Swedish study found that self-stigma significantly predicted the intention to seek online help among college students and primary care patients [39]. The inconsistency in previous literature suggests that the dynamics of self-stigma in digital contexts may differ from traditional settings and warrant further investigation.

The absence of a significant association between self-stigma and choosing online therapy in the logistic regression in this study might be attributed to several factors. First, other variables such as perceived effectiveness of online therapy, accessibility, and technological proficiency may play a more substantial role in the decision-making process. Second, the relationship between self-stigma and help-seeking behavior is complex and may be influenced by additional factors, such as cultural norms and individual differences [74,75].

The study showed that openness was the only trait negatively associated with self-stigma in both the online and offline groups. Self-stigma occurs when individuals internalize prejudiced beliefs prevalent in society, leading to feelings of shame and reduced help-seeking behavior [71]. Research demonstrates that openness to experience is associated with cognitive flexibility and a lower tendency to accept rigid stereotypes or prejudices, which may in turn reduce the internalization of stigmatizing beliefs about mental health and lead to lower levels of self-stigma [76,77].

Additionally, individuals with high openness are more willing to disclose psychological distress, as they tend to view self-exploration and emotional vulnerability as opportunities for personal growth rather than sources of self-stigma [78]. In Egyptian and broader Arab communities, individuals often express psychological distress through physical symptoms rather than verbalizing emotional difficulties, in an effort to avoid the perceived weakness and social stigma associated with mental illness and help seeking, which is often viewed as socially unacceptable. Mental health issues are frequently associated with shame, leading families to conceal a relative’s psychiatric diagnosis for fear of damaging their social standing or marriage prospects. This stigma is further reinforced by cultural norms that place a high value on endurance and emotional restraint, discouraging open discussion of mental health struggles [40,79].

There are inconsistent results regarding openness and self-stigma, with previous research showing no direct association between them [74,75]. The complex relationship between self-stigma of seeking help and personality traits is rarely studied in the context of telepsychiatry. Therefore, further investigation is needed to understand how factors associated with self-stigma operate across online and offline settings. Understanding these factors associated with self-stigma is essential due to the high levels of mental health stigma, which pose a significant barrier to help seeking, especially in Egypt and the Arab world [80].

### Implications and Future Research

This study has significant implications for clinical practice and underscores the need for future research. Understanding the personal and psychological factors that influence the preference for online therapy can help mental health professionals tailor their services to better meet the needs of patients. The significant differences in self-stigma and personality traits between online and offline help seekers highlight the importance of exploring these factors further, particularly as online therapy emerges as an increasingly vital modality for mental health support, likely to expand in popularity and accessibility. Consequently, future research should focus on replicating these findings among diverse populations and with larger sample sizes to enhance their generalizability. Importantly, this study opens the door for investigating differences between actual offline and online help seekers, necessitating further studies to address the existing knowledge gap surrounding help-seeking behaviors. By expanding this research, we can gain deeper insights into the complexities of mental health service use, ultimately contributing to a more comprehensive understanding of the motivations behind choosing different modalities for support.

### Strengths and Limitations

This study aimed to compare actual online and offline mental health help seekers in terms of sociodemographic factors, personality traits, and self-stigma, rather than merely assessing preferences and intentions, by focusing on individuals who sought mental health care. The findings serve as preliminary data for further research and contribute to the representation of Egyptian and Arabic populations in the literature. Despite its strengths, the study has several limitations. The relatively small sample size and recruitment from one online platform and a few private clinics may restrict the generalizability of the findings to the broader population of mental health service users. Additionally, the use of convenience sampling may introduce selection bias, as participants who engage with the study may differ systematically from those who do not. Furthermore, conducting the study in only one country limits the ability to draw broader conclusions applicable to diverse cultural contexts, highlighting the need for future research to replicate these findings in different settings and populations.

### Conclusions

In conclusion, this study provides key insights into the differences between individuals who prefer online versus offline mental health services. Logistic regression analysis identified several significant factors associated with online service preference. The strongest factor was a history of online psychiatric consultation, highlighting the role of previous exposure in shaping treatment choices. Employment status and ease of internet access were also positively associated with choosing online therapy. In contrast, a history of in-person consultations and higher levels of openness and conscientiousness were associated with a preference for offline services. Higher agreeableness showed a marginal association with choosing online therapy. These findings suggest that treatment modality preferences are influenced by a complex interplay of previous experiences, sociodemographic factors, and personality traits. Tailoring mental health interventions to these factors may enhance service accessibility and engagement. Future research should aim to validate these findings in more diverse populations and explore additional cultural and structural influences.

## Appendix

Multimedia Appendix 1 Supplementary figures and tables.

## Abbreviations

CHERRIES: Checklist for Reporting Results of Internet E-Surveys
OR: odds ratio
SSOSH: Self-Stigma of Seeking Help

## Data Availability

The datasets used and/or analyzed during this study are available from the corresponding author on reasonable request.
