# Supplementary material for: Understanding the Differences Between Online and Offline Mental Health Help Seekers: Cross-Sectional Comparative Study
Source: JMIR Hum Factors. 2025 Nov 7;12:e69305. doi: 10.2196/69305 (PMC12639346; doi:10.2196/69305)
Supplement: Multimedia Appendix 1 [file humanfactors_v12i1e69305_app1.docx]

**Figure S1.** Reasons for choosing online therapy.

**
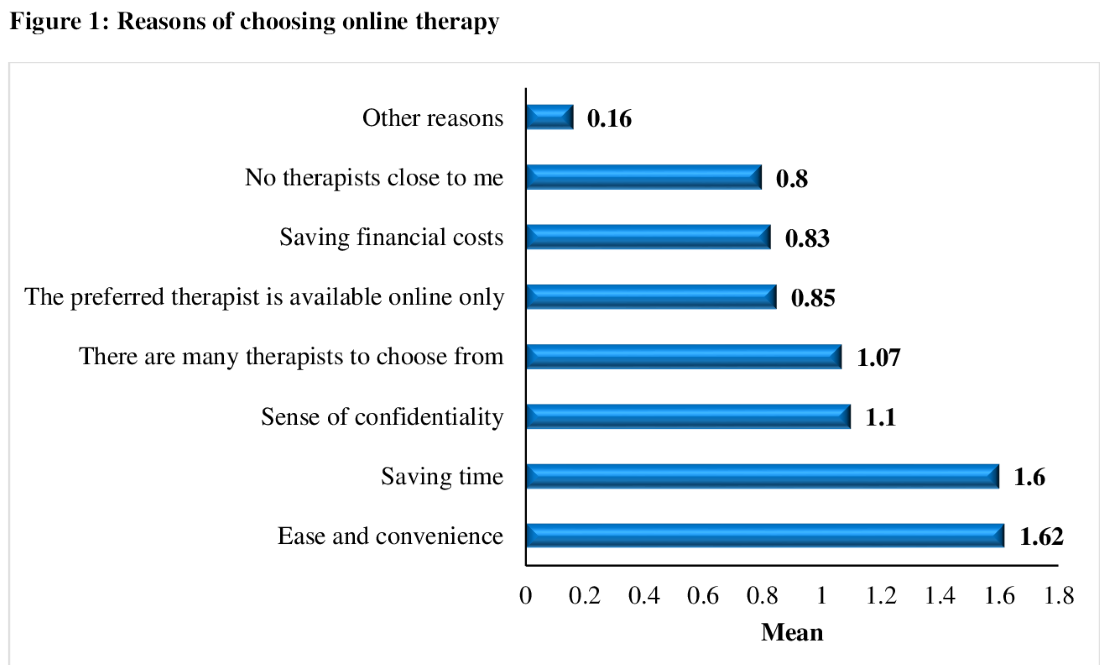
**

**Figure S2:** Recruitment flowchart. (OLD S1)


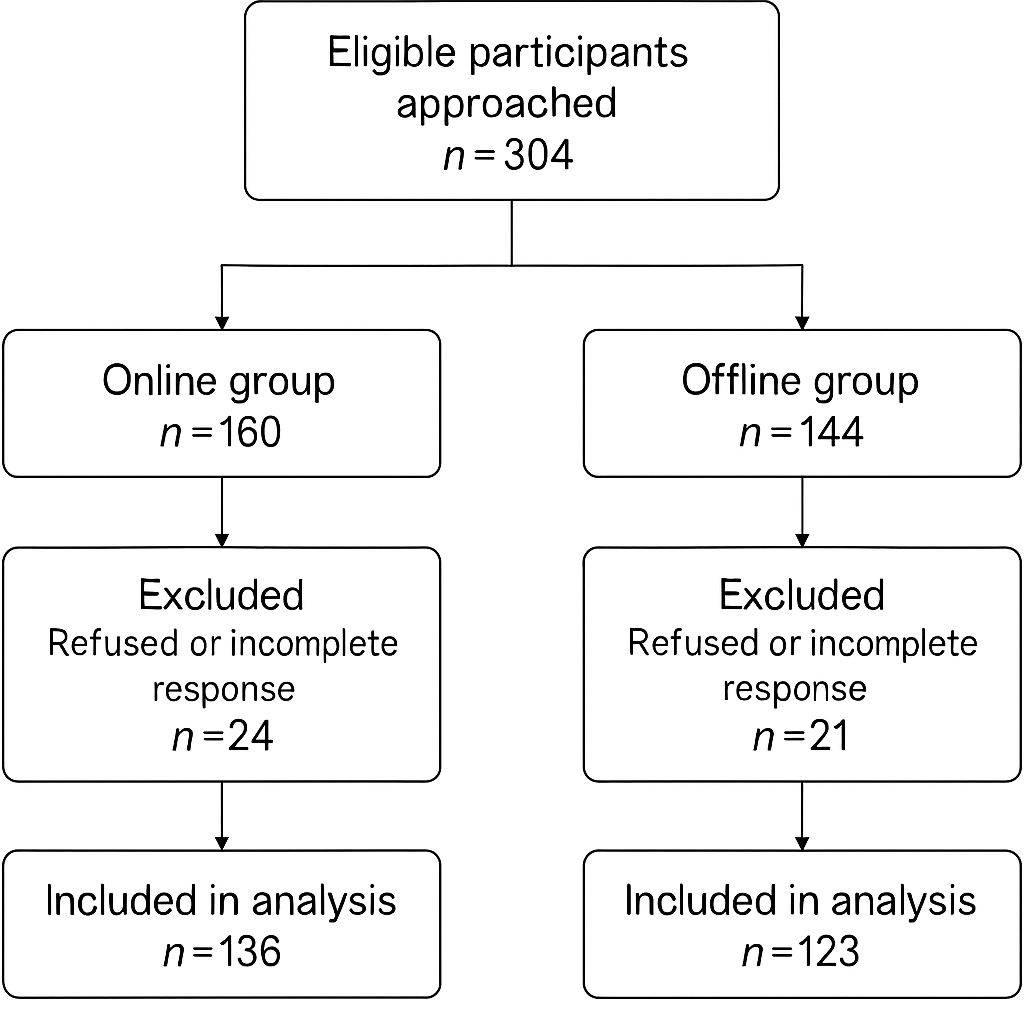


**Figure S3:** Frost plot of the significant factors affecting online patients. (OLD S2)

**Table S1.** False discovery rate.

| **Factor** | **Original P value** | **Critical Value** | **Benjamini-Hochberg Adjusted P value** | **Significant using an FDR of 0.05?** |
| --- | --- | --- | --- | --- |
| SSOSH | 0.0001 | 0.002083333 | 0.0006 | Yes |
| psychiatric online visit | 0.0001 | 0.004166667 | 0.0006 | Yes |
| working status | 0.0001 | 0.00625 | 0.0006 | Yes |
| psychiatric clinic visit | 0.0001 | 0.008333333 | 0.0006 | Yes |
| internet ease access | 0.001 | 0.010416667 | 0.0048 | Yes |
| education | 0.003 | 0.0125 | 0.010285714 | Yes |
| Consciousness | 0.003 | 0.014583333 | 0.010285714 | Yes |
| highest education | 0.022 | 0.016666667 | 0.066 | No |
| internet access | 0.029 | 0.01875 | 0.077333333 | No |
| residence | 0.079 | 0.020833333 | 0.1896 | No |
| extraversion | 0.103 | 0.022916667 | 0.224727273 | No |
| breadwinner | 0.133 | 0.025 | 0.252 | No |
| sex | 0.139 | 0.027083333 | 0.252 | No |
| receiving charity | 0.147 | 0.029166667 | 0.252 | No |
| working sector | 0.227 | 0.03125 | 0.3632 | No |
| income | 0.247 | 0.033333333 | 0.3705 | No |
| marital status | 0.321 | 0.035416667 | 0.453176471 | No |
| working place | 0.407 | 0.0375 | 0.542666667 | No |
| agreeableness | 0.526 | 0.039583333 | 0.664421053 | No |
| opennes | 0.66 | 0.041666667 | 0.762285714 | No |
| neuroticism | 0.667 | 0.04375 | 0.762285714 | No |
| age | 0.777 | 0.045833333 | 0.847636364 | No |
| working condition | 0.844 | 0.047916667 | 0.880695652 | No |
| working time | 0.89 | 0.05 | 0.89 | No |

**Table S2.** Variance inflation factor for multicollinearity of factors used in logistic regression.

| **Independent variables** | **Tolerance** | **VIF** |
| --- | --- | --- |
|  |  |  |
|  |  |  |
| **Education level** | 0.684 | 1.462 |
| **Working status** | 0.692 | 1.444 |
| **Having internet** | 0.851 | 1.175 |
| **Ease access to internet** | 0.856 | 1.168 |
| **History of psychiatric consult in a clinic** | 0.909 | 1.100 |
| **History of psychiatric consult online** | 0.817 | 1.224 |
| **Extraversion** | 0.776 | 1.289 |
| **Neuroticism** | 0.837 | 1.194 |
| **Agreeableness** | 0.711 | 1.406 |
| **Openness** | 0.659 | 1.517 |
| **Consciousness** | 0.735 | 1.360 |
| **SSOSH** | 0.779 | 1.284 |

**Interpretation:**

**Tolerance** = 1 / VIF

**VIF > 5** indicates moderate multicollinearity.

**VIF > 10** is often considered serious multicollinearity.
